# Supply-side dimensions and dynamics of integrating HIV testing and counselling into routine antenatal care: a facility assessment from Morogoro Region, Tanzania

**DOI:** 10.1186/s12913-015-1111-x

**Published:** 2015-10-04

**Authors:** Selena J. An, Asha S. George, Amnesty E. LeFevre, Rose Mpembeni, Idda Mosha, Diwakar Mohan, Ann Yang, Joy Chebet, Chrisostom Lipingu, Abdullah H. Baqui, Japhet Killewo, Peter J. Winch, Charles Kilewo

**Affiliations:** Department of International Health, International Center for Maternal and Newborn Health, Johns Hopkins Bloomberg School of Public Health, 615 N Wolfe Street, Baltimore, MD 21205 USA; Department of Epidemiology and Biostatistics, Muhimbili University of Health and Allied Sciences, P.O. Box 65015, Dar es Salaam, Tanzania; Department of Behavioural Sciences, Muhimbili University of Health and Allied Sciences, P.O. Box 65015, Dar es Salaam, Tanzania; Department of Obstetrics and Gynecology, Muhimbili University of Health and Allied Sciences, P.O. Box 65015, Dar es Salaam, Tanzania; Jhpiego, Dar es Salaam, Tanzania

**Keywords:** ANC, HIV testing and counselling, Infrastructure, Human resources, Drugs, Equipment, Facility assessment, Integration

## Abstract

**Background:**

Integration of HIV into RMNCH (reproductive, maternal, newborn and child health) services is an important process addressing the disproportionate burden of HIV among mothers and children in sub-Saharan Africa. We assess the structural inputs and processes of care that support HIV testing and counselling in routine antenatal care to understand supply-side dynamics critical to scaling up further integration of HIV into RMNCH services prior to recent changes in HIV policy in Tanzania.

**Methods:**

This study, as a part of a maternal and newborn health program evaluation in Morogoro Region, Tanzania, drew from an assessment of health centers with 18 facility checklists, 65 quantitative and 57 qualitative provider interviews, and 203 antenatal care observations. Descriptive analyses were performed with quantitative data using Stata 12.0, and qualitative data were analyzed thematically with data managed by Atlas.ti.

**Results:**

Limitations in structural inputs, such as infrastructure, supplies, and staffing, constrain the potential for integration of HIV testing and counselling into routine antenatal care services. While assessment of infrastructure, including waiting areas, appeared adequate, long queues and small rooms made private and confidential HIV testing and counselling difficult for individual women. Unreliable stocks of HIV test kits, essential medicines, and infection prevention equipment also had implications for provider-patient relationships, with reported decreases in women’s care seeking at health centers. In addition, low staffing levels were reported to increase workloads and lower motivation for health workers. Despite adequate knowledge of counselling messages, antenatal counselling sessions were brief with incomplete messages conveyed to pregnant women. In addition, coping mechanisms, such as scheduling of clinical activities on different days, limited service availability.

**Conclusion:**

Antenatal care is a strategic entry point for the delivery of critical tests and counselling messages and the framing of patient-provider relations, which together underpin care seeking for the remaining continuum of care. Supply-side deficiencies in structural inputs and processes of delivering HIV testing and counselling during antenatal care indicate critical shortcomings in the quality of care provided. These must be addressed if integrating HIV testing and counselling into antenatal care is to result in improved maternal and newborn health outcomes.

## Background

More than two-thirds of new adult HIV infections and more than 80 % of new child infections in 2011 occurred in sub-Saharan Africa [1]. In addition, almost 90 % of HIV-related maternal deaths occurred in sub-Saharan Africa in 2012 [2]. Integration of HIV into reproductive, maternal, newborn and child health (RMNCH) services is therefore an important process addressing the disproportionate burden of HIV in sub-Saharan Africa.

Integration can be conceptualized as the process of incorporating a vertical or stand-alone intervention into a health system across different dimensions or levels [3]. According to the World Health Organization (WHO), “integration of HIV interventions into maternal, newborn and child health (MNCH) services involves the reorganization and reorientation of health systems to ensure the delivery of a set of essential interventions for HIV prevention, treatment and care as part of the continuum of care for women, newborn, children and families” [4]. The reorganization aims to increase coverage of interventions, reduce stigma, and enhance client retention, which have been achieved in some programs integrating HIV services into maternal and child health services [5, 6]. However, in certain contexts the effectiveness and responsiveness of service delivery may be further constrained with integration [5], due to unmanaged increases in the workload of service providers [5] and loss of confidentiality for women seeking care [7, 8].

The integration of HIV testing and counselling into routine antenatal services is a strategy to maximize coverage of pregnant women in prevention of mother-to-child transmission (PMTCT) interventions. In Tanzania, PMTCT policy and programming have moved towards being integrated with RMNCH services over time. PMTCT started as a pilot program during 2000–2002, after which the national program and first guidelines were established in 2004 [9]. In these guidelines, HIV services were provided to pregnant women on an “opt in” basis, where testing, counselling and treatment were done outside of the reproductive and child health (RCH) clinics in separate care and treatment centers (CTC). In 2007, Tanzania developed a national PMTCT scale up plan (2007–2012) with population-based targets that articulated a strategy to deliver a comprehensive package of PMTCT interventions. In this plan, the adoption of provider-initiated testing and counselling strategy shifted the PMTCT program to an “opt out” intervention. While HIV testing and counselling were integrated into RCH services, subsequent HIV-related care for HIV-infected pregnant women remained in CTCs [10, 11]. In 2013, Tanzania moved to Option B+, where lifelong treatment is given to all HIV-infected pregnant and lactating mothers, regardless of CD4 count and WHO clinical disease stage. These services are fully integrated into the RCH services package [12] (Table 1).

**Table 1:** PMTCT policies in Tanzania

Year	Source	Policy content
2000–2002	Pilot PMTCT Program	• Short course regimen for preventing mother-to-child transmission in four referral hospitals and one regional hospital
		• Use of AZT short course from 36 weeks to delivery
2004	First national PMTCT guidelines for scale up	• Scale up from 5 pilot testing sites to the whole country (1347 sites across the country by 2006)
		• sdNVP during labor and delivery
2007	Second national PMTCT guidelines for scale up	• Provider initiated testing and counselling in antenatal visits in an “opt out” system
		• PMTCT remained in parallel to Care and Treatment Centers (CTC), where eligible mothers received care
		• Change of regimen from sdNVP to AZT from 28 weeks of pregnancy until labor and delivery for PMTCT
2011	Third national PMTCT guidelines for scale up	• Tanzania adopts option A of 2010 WHO guidelines (use of ARV drugs for treating pregnant women and preventing mother-to-child transmission of HIV)
		• Engagement with, testing of, and counselling partners at health facilities
		• PMTCT program expanded to 3420 sites in the country
2013	Fourth national PMTCT guidelines Option B/B+	• All HIV-infected pregnant and lactating mothers, regardless of CD4 count, eligible for lifelong treatment with antiretroviral drugs
		• Care and treatment integrated into RCH wards

As mentioned previously, antenatal care serves as a critical platform into which HIV testing and counselling were integrated in 2007 in Tanzania [13–15]. Antenatal care is potentially a universal platform for pregnant women, as 96 % of women received at least one antenatal care visit from a skilled provider during pregnancy in mainland Tanzania in 2010 [16]. However, only 15 % of women made their first antenatal care visit during the first trimester, and only 43 % completed the four visits recommended by the focused antenatal care (FANC) guidelines (Table 2) [16].

**Table 2:** FANC services in Tanzania

Focused antenatal care checklist				
Parameter	First visit <16 weeks	Second visit 20–24 weeks	Third visit 28–32 weeks	Fourth visit 36 weeks
Laboratory investigations, blood				
Hemoglobin	✓	✓	✓	✓
Grouping and rhesus factor	✓			
RPR	✓			
First HIV testing	✓			
Client education and counselling (for the couple)				
Process of pregnancy and complications	✓	✓	✓	✓
Diet and nutrition	✓	✓	✓	✓
Rest and exercise in pregnancy	✓	✓	✓	✓
Personal hygiene	✓			
Danger signs in pregnancy	✓	✓	✓	✓
Use of drugs in pregnancy	✓	✓	✓	✓
Effects of STI/HIV/AIDS	✓	✓	✓	✓
Voluntary counselling and testing for HIV	✓			
Care of breasts and breast feeding	✓			✓
Symptoms/signs of labor			✓	✓
Plans of delivery (emergency preparedness, place of delivery, transportation, financial arrangements)	✓	✓	✓	✓
Plans for postpartum care			✓	✓
Family planning			✓	✓
Harmful habits (e.g. smoking, drug abuse, alcoholism)	✓	✓	✓	✓
Schedule of return visit	✓	✓	✓	✓

Source: Adapted from Von Both C, Fleβa S, Makuwani A, Mpembeni R, Jahn A. How much time do health services spend on antenatal care? Implications for the introduction of the focused antenatal care model in Tanzania. BMC Pregnancy Childbirth. 2006;6(22)

Recognizing that integration is a broad and complex topic, and considering the known limitations of the antenatal care platform as well as the context of evolving HIV policies in Tanzania, we focus on the supply-side dimensions and dynamics of integrated HIV testing and counselling during routine antenatal care in Morogoro Region, Tanzania. Supply-side dimensions, according to the quality of care literature, consist of structural elements, the context in which health care is provided, health care processes that support care seeking and provision, and health outcomes [17, 18]. Shigayeva further identifies the following as critical structural inputs: infrastructure; laboratories (space); drugs, medical supplies and technologies (supplies); and availability of human resources (staffing) [19]. We previously reported on the demand-side dynamics related to the integration of HIV testing and counselling into antenatal care [20]. By focusing on supply-side dimensions in this paper, we complement previous research and highlight critical service delivery dynamics that must be addressed as Tanzania moves towards further integration under Option B+.

## Methods

### Study site

Populated with 45 million people and located in east Africa, Tanzania is a low-income country with a per capita gross national income of $540 US dollars [21, 22]. In 2012, 10 % of the Tanzanian government budget was spent on health, in comparison to the Abuja Declaration’s target of 15 % for African Union countries [23, 24]. There are 7 hospital beds and 2 physicians, nurses, and midwives per 10,000 population, much lower than the WHO-recommended minimum of 23 doctors, nurses, and midwives per 10,000 population necessary for the provision of essential maternal and child health services [25, 26]. The Tanzanian health system is structured as a pyramid, with village health service at the bottom, followed by dispensary services, health center services, district hospitals, regional hospitals, and referral/consultant hospitals at the top [27]. Village health services consist of preventive care provided by volunteers who have received training [27]. Dispensaries are the first level of primary care with outpatient services and services for uncomplicated deliveries. At the next level, health centers provide inpatient and outpatient care, including labor and delivery services; receive referrals from lower levels of the health system; and conduct outreach activities that include preventive services [28]. The Tanzanian Ministry of Health and Social Welfare (MoHSW) recommended staffing requirements indicate that each health center should have at least one assistant medical officer, three clinical officers, one registered nurse, four enrolled nurses, four medical assistants, and one laboratory technician [29]. Hospitals at higher levels of the health system have greater clinical capability, including surgical care, in addition to testing and diagnostic services such as radiology and laboratory.

Located about 200 km outside of Dar es Salaam in eastern Tanzania, Morogoro Region is one of 30 administrative Regions in the country [22]. With a population of 2 million and a population density of 31 inhabitants per square kilometer, Morogoro Region is among Tanzania’s largest and least densely populated regions. Regional averages in Morogoro for education, poverty and health care seeking are similar to national averages. Seventy three percent (73 %) of women and 85 % of men in Morogoro Region are able to read, in comparison to 72 and 82 % nationally [22]. According to the Tanzania Demographic and Health Survey in 2010, 23 % of women in Morogoro reported having at least one problem in accessing health care [16]. Five percent (5 %) of women of reproductive age and 2 % of men between the ages of 15–49 in Morogoro are HIV-positive, and 67 % of women of reproductive age and 50 % of men between 15 and 49 in Morogoro have ever tested for HIV [30].

### Study design

This exploratory study of the supply-side dimensions supporting HIV testing and counselling in antenatal care in Tanzania formed part of a larger three-year evaluation of a maternal, newborn, and child health program implemented by the Tanzanian MoHSW with technical assistance from Jhpiego in Morogoro Region. All 18 primary care health centers in four out of six districts in Morogoro Region (Kilosa,^1^ Morogoro District Council, Mvomero, and Ulanga) were chosen for a cross-sectional health facility assessment (Table 3). Morogoro Municipal Council was excluded as it has the regional hospital and thus differs from other parts of Morogoro Region. Kilombero district was excluded since it has a different community health worker program supported by the Ifakara Health Institute.

**Table 3:** Characteristics of 18 primary care health centers

	Urban health centers (*N* = 1)	Suburban health centers (*N* = 5) (mean/median/range)	Rural health centers (*N* = 12) (mean/median/range)
Number of Years in Service	33	33/34/0–51	26/28/3–53
Catchment Area, Number of Villages	5	9/6/6–19	5/4/2–14
Catchment Area, Population	35,262	25,706/20,489/12,986–54,000	9,828/9,377/4,470–14,157
Number of RCH Providers	7	12/8/4–24	11/12/4–20
Medical officers (MoHSW recommendation: 0)	0	0/0/0–1	0/0/0–1
Assistant medical officers (MoHSW recommendation: 1)	3	1/1/0–2	1/1/0–5
Clinical officers (MoHSW recommendation: 3)	10	3/2/1–5	3/3/0–6
Assistant clinical officers (MoHSW recommendation: 0)	0	1/1/0–2	0/0/0–2
Registered nurses (MoHSW recommendation: 1)	0	3/3/0–7	2/2/0–4
Enrolled nurses (MoHSW recommendation: 4)	15	4/3/2–7	4/5/1–7
Medical attendants (MoHSW recommendation: 4)	7	9/8/7–11	4/3/0–9

The facility assessment included quantitative and qualitative data collection activities (Table 4). At each health center, an interview with the provider-in-charge was conducted, which included checklists for supplies and equipment. In addition, direct observations were carried out using standardized checklists drawn from Service Provision Assessment (SPA) and Standards-Based Management and Recognition (SBMR) tools [31, 32]. In the event of drug stock-outs, the health center in-charge and/or attending pharmacist were asked to provide details on the duration and/or ever-availability of the product in question.

**Table 4:** Data sources included in MNCH facility survey

Data source	Sampling	Final sample
Facility observation checklists and interviews with facility in-charge	Census of health centers	18
ANC provider interviews, quantitative	Sub-analysis of 88 RCH providers interviewed based on availability on day of visit and provision of antenatal care in preceding 7 days	65
ANC provider interviews, qualitative	Sub-sample of 88 RCH providers interviewed based on availability on day of visit, receipt of Jhpiego PNC training, and length of service at the facility, average of 3 per facility	57
ANC sessions observed	Quota based on availability on day of visit, average of 10 per facility, total approved target sample of 240	203; 8 refusals

Pregnant women were selected based on quota sampling with a planned total of 240. On average, the first ten eligible women attending routine antenatal services at each health center on the day of the research team’s visit were recruited for direct observation of their antenatal session and exit interviews.

At each health center, of the approximately 20 total providers on the roster, five to ten were present during the data collection shift of 8 am–6 pm and a subset of these were RCH providers. Selection of providers was purposive and included only RCH staff who reported providing antenatal and postnatal care services, were present in the health center during one of the two days of observation, and consented to be interviewed. At least five RCH providers from each health center were enrolled in the study. Due to the broader evaluation goals, quantitative data including demographic information, education and training, knowledge of counselling messages, reported counselling practices, supervision, and compensation were collected from all five providers. Additional quantitative data about care delivery processes, including provision of counselling, were collected during direct observations of antenatal consultations. Qualitative in-depth interviews of 30 minutes to one hour in duration were administered to a sub-sample of three RCH providers per health center, chosen based on their Jhpiego training in postnatal services, provision of maternal and newborn health services, and years of service. Qualitative provider interviews (*N* = 57) covered antenatal care and postnatal care service utilization, integration of family planning and HIV services, and linkages to other levels of the health system. Providers were identified by their codes, which consisted of the facility number and the number from the employee list provided by the facility in-charge (i.e. facility number-employee number, 01–01).

### Data collection

A team of six research assistants, including two trained in social sciences, two medical doctors, and two quantitative researchers, received training from study investigators over a one-week period in mid-September 2012. During training and pilot testing, research assistants who observed antenatal sessions practiced until they received a 95 % reliability score. Pilot testing was followed by data collection in late September to early December 2012 in Morogoro, with data collected over two to three days in each health center.

At each health center, study personnel visited the health center in-charge to brief him or her on data collection objectives and ascertain the days antenatal and postnatal services were provided to coordinate timing for data collection. At many health centers, antenatal services and HIV testing and counselling were done only on a weekly basis, often corresponding with “Market Day” or other non-health activities.

Data quality was ensured by two field-based supervisors who provided overarching support to field implementation, including review of completed instruments and daily debriefings. Completed and supervisor-checked questionnaires were sent to Muhimbili University of Health and Allied Sciences (MUHAS) in Dar es Salaam for data entry and cleaning. Provider qualitative interviews were digitally recorded, transcribed, and translated into English. Debriefs of notes taken by research assistants were conducted daily, at midpoint and at endpoint of data collection. These debriefs allowed for a quality review of the qualitative data and discussions about emerging themes and instances of uncertainty where negative or contradictory data needed further exploration. Through these debriefs, the team also triangulated data by sources (providers and women), investigators (two research assistants conducting interviews with each type of respondents), and methodology (qualitative and quantitative information from interviews, facility assessment surveys, health center records, and observations) to ensure reliability and validity of results. After the midpoint debrief, revised interview guides focusing on emerging themes were implemented for the last seven health centers visited by the research team.

### Analysis

Thematic qualitative data analysis was undertaken from a database coded and organized by Atlas.ti [33]. Codes were derived by consensus through team discussion based on the categories and the structure of the interview guide and on themes that emerged from the daily debriefings among research assistants and supervisors as well as midpoint and endpoint debriefings with the larger team. In the discussions and analysis, we attempted to balance the perspectives of both providers and pregnant women.

Quantitative data were entered and cleaned using Epi Info software having built in range and consistency checks. Descriptive analyses were performed using Stata 12.0 [34] and results examined by groupings of health centers based on their administrative designation of urban, suburban, and rural settings. Summary composite scores were calculated to indicate facility readiness to deliver care. These composite scores are the sum of relevant individual indicators, broken down into four domains: 1) HIV diagnostic and treatment services, which included the presence of laboratories and CTCs; 2) waiting and registration area, which included the presence of waiting area, presence of covering or roofing over the waiting area, and whether the registration/waiting area was well-ventilated; 3) furniture, which included the presence of at least one desk and at least one chair for provider and of at least one chair for patient as well as sufficient chairs and space for one companion of each patient; and 4) counselling area, which included the presence of dividing curtain or screen, whether the group counselling area was well-ventilated, and presence of sufficient space for pregnant women to walk. Indicators were weighted equally and summary composite scores calculated by the probability-weighted average for each domain.

Preliminary findings were shared with key decision makers in Tanzania belonging to MoHSW and Jhpiego for their feedback and review prior to publications being drafted.

### Ethical approval

The study received ethical approval from the MUHAS and Johns Hopkins School of Public Health Institutional Review Boards. Permission to conduct the study was obtained from MoHSW and from the region and district administration authorities. Individual written consents were obtained from the study participants prior to their participation in the study.

## Results

In terms of supply-side structural inputs and processes supporting HIV testing and counselling in routine antenatal care provided at health centers, we first review results regarding space or physical infrastructure and the availability of supplies (drugs and equipment). We then describe issues related to staffing, including health worker respondent characteristics and their availability. We also detail their provision of services, including their knowledge and delivery of HIV counselling messages during antenatal care. We conclude with results that further describe the context and content of HIV counselling during antenatal care to provide a more complete understanding of supply-side elements of delivering such services.

### Infrastructure

**Fig. 1:**
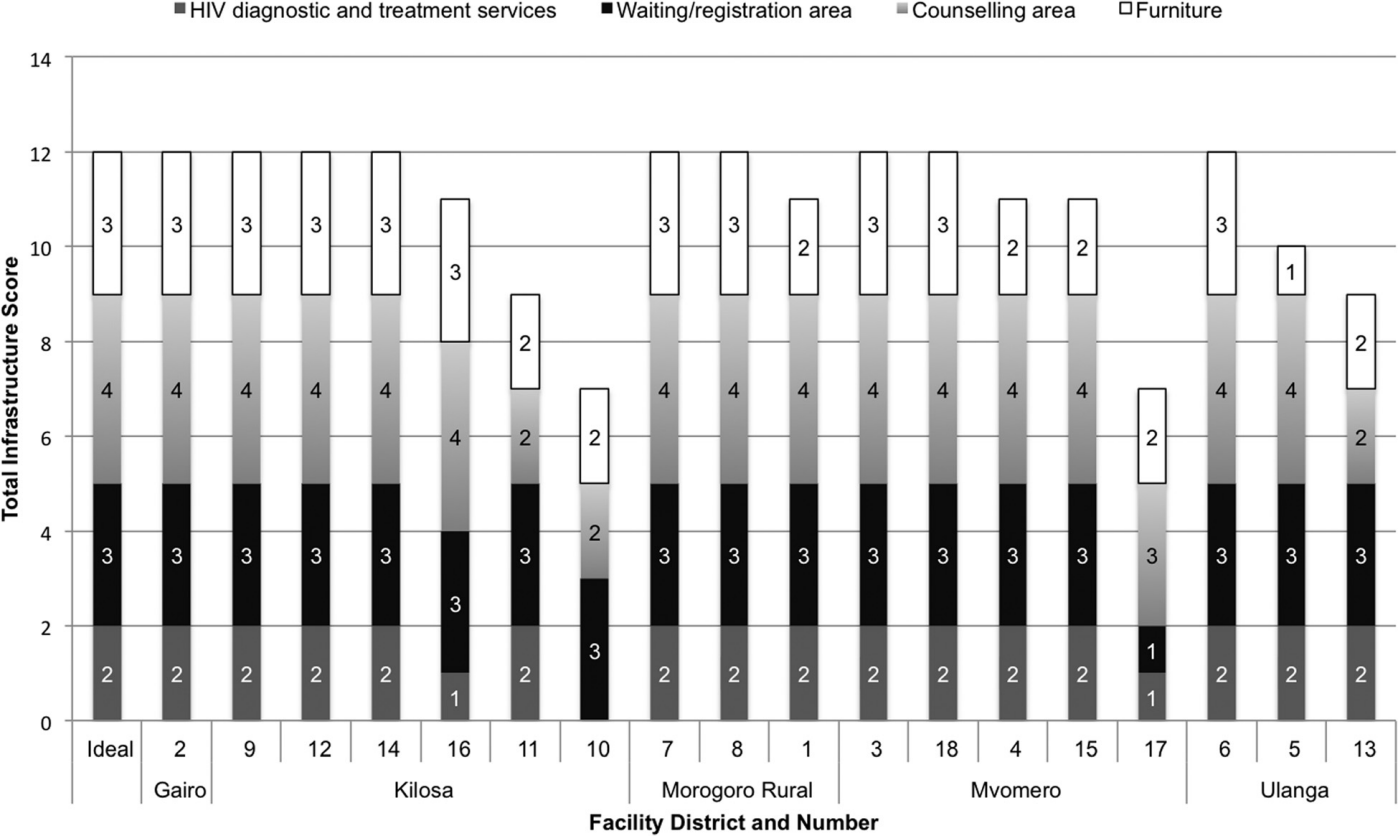
Availability of Infrastructure. The health infrastructure composite scores include **a**) HIV diagnostic and treatment services (laboratory, presence of CTC); **b**) waiting and registration area (waiting area, covered or roofed waiting area, well-ventilated registration/waiting area); **c**) counselling area (dividing curtain or screen, well-ventilated group counselling area, and sufficient space for pregnant women to walk); **d**) furniture (at least one desk and at least one chair for provider, at least one chair for patient; sufficient chairs and space for one companion of each patient)

Just over half of the health centers had a complete composite score taking into consideration four domains: 1) presence of HIV diagnostic and treatment services, 2) availability of waiting and registration area, 3) availability of counselling area, and 4) availability of furniture (Fig. 1). Spacing was judged based on the availability of any space, not the extent of the space. Variations in infrastructural deficiencies among health centers did not differ significantly by groupings of urban, suburban, and rural health centers. Two outlier health centers (one designated as rural and one designated as suburban) with the lowest composite scores were both undergoing upgrading from dispensaries to health centers at the time of data collection.

Of the health centers with deficiencies, three facilities lacked either a laboratory or a CTC, or both. One health center did not have a well-ventilated waiting/registration area or group counselling area. While all health centers had a well-ventilated group sitting area, researchers observed that two health centers did not have sufficient open space in which pregnant women could walk while waiting for routine antenatal services. While all health centers had at least one chair for women, eight health centers lacked a desk for providers and one health center in addition lacked at least one chair for providers.

For just over half of the health centers, space was estimated to be sufficient based on the composite indicators of availability mentioned above; however, the research team observed crowded waiting areas at most health centers. In addition, health workers commented on the lack of space to provide private and confidential services to HIV-positive pregnant women (Provider 04–12, enrolled nurse). Provider 07–09, an enrolled nurse, said:

*The room is too small… It’s hard to give counselling here, share results, and you have to come inside… It would have been better if we had a big room, with space for HIV, PMTCT counselling, space for testing… [A patient] can come and find [the] place is full of people.*

### Supplies

In Tanzania, the Medical Stores Department (MSD), a semiautonomous organization under the MoHSW, procures and distributes all equipment and supplies [35]. At the time of research, facility in-charges order the supplies and equipment each month based on projections from previous months. The MSD then orders and distributes supplies by zone. For Morogoro, the supplies are ordered and come from the Coastal zone located in Dar es Salaam. At the time that the research team was collecting data, the zonal MSD took supplies to the District Medical Officer (DMO), who distributed the drugs and supplies to health facilities according to the requisition orders from each facility. Today, drugs, including HIV drugs, are ordered on a quarterly basis, and zonal MSD take drugs either directly to the health facility or to the DMO. Initial funds for HIV drugs are provided by external donors, and the MSD handles distribution of the drugs throughout Tanzania.

**Fig. 2:**
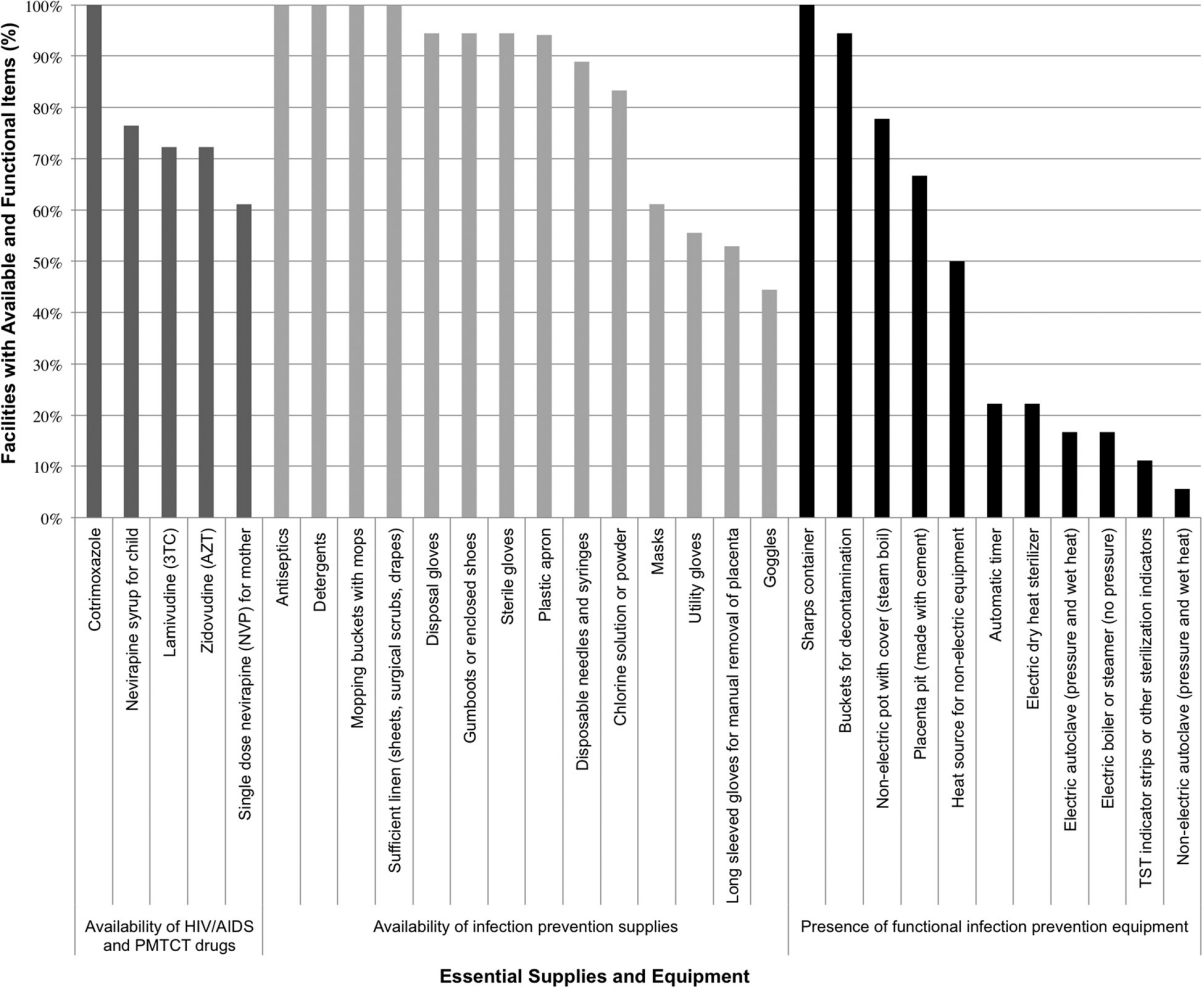
Availability of functional essential supplies and equipment for delivery of integrated HIV/ANC services

At the time of the research team’s visit, six (33 %) health centers could not undertake HIV testing. One health center attributed this to the lack of equipment such as gloves, while the other five attributed this to the lack of supplies such as HIV test kits. In addition to test kits, there was also a shortage of some types of drugs for HIV and PMTCT. Of the six drugs on the facility assessment checklist, single dose nevirapine (sdNVP) for the mother had the most unreliable supply: seven out of the 18 (39 %) health centers did not have a continuous supply in the 30 days preceding the research team’s visit. One health center reported that the drug had been out of stock for six months. Five health centers reported zidovudine (AZT) and lamivudine (3TC) out of stock and four reported lack of nevirapine syrup for children (Fig. 2). Variations in levels of HIV drug supplies and equipment did not differ by the location of the health center in urban, suburban, or rural settings. One outlier facility in a rural area had the lowest levels of stock for all three categories included under supplies: HIV drugs (four out of five was out of stock), infection prevention supplies (seven out of 14 were unavailable), and infection prevention equipment (nine out of 11 were not present). The low levels of supplies at this facility can be attributed to its recent upgrade from a dispensary to a health center at the time of the research team’s visit.

In coping with these shortages, pregnant mothers at one health center were asked to bring gloves, typically during labor and delivery, to prevent the possibility of infection:

*I do not want to infect you or for you to infect me either.* (Provider 07–09, enrolled nurse)

Apart from increasing the burden of seeking services on women, health workers noted with frustration that the lack of supplies also led to missed opportunities:

*It’s just that missing supplies made these people not able to return …you know…when you give someone* [services], *you are supposed to test her immediately… If you delay, she might slip* [out of the health system]*.* (Provider 18–08, registered nurse)

Women had varying responses to missing supplies. One provider discussed women’s reactions to arriving at the health center and finding that the HIV test could not be performed due to stock-outs as thus:

*Some women keep on reminding* [their providers during subsequent antenatal visits] *that they didn’t take an HIV test during the first visit at the clinic because there were no facilities…for those* [women] *who didn’t like to test, they intentionally take advantage. (*Provider 07–06, enrolled nurse)

In addition to missed opportunities to engage with pregnant women and provide HIV counselling, providers also saw the lack of supplies as undercutting their relationship with their patients and the reputation of health centers in the community. HIV test kit stock-outs were described as an “embarrassment”, and that:

S*ome* [women] *ask us why others did not get tested and we did? So it’s true everything we do here gets back to the community.* (Provider 02–27, enrolled nurse)

### Staffing

#### Characteristics of health worker respondents

The mean age of antenatal providers interviewed was 39 years, and 78 % were women. Almost half of the providers were married or co-habiting; 38 % were single; and the remaining 11 % were widowed, divorced, or separated. They worked a mean of 14 years as a health worker, including 6 years at the health center where the interview was conducted (Table 5). Although 71 % were enrolled and registered nurses, 15 % were medical assistants and 3.1 % were health assistants. According to guidelines, neither medical nor health assistants are qualified to provide antenatal care.

**Table 5:** Characteristics of antenatal care providers interviewed (*N* = 65)

Age (mean/median/range)	39.2/37.9/13–60
Female	78.5 %
Marital status	
Married/Co-habitating	49.2 %
Single	38.5 %
Widowed/divorced/separated	10.8 %
Designation	
Assistant medical officer, 5 years of clinical training	1.5 %
Clinical officer, 3 years of clinical training	6.2 %
Assistant clinical officer, 3 years of clinical training	1.5 %
Assistant nursing officer	1.5 %
Registered nurse, 4 years of nursing training	15.4 %
Enrolled nurse, 3 years of nursing training	55.4 %
Medical assistant, secondary school	15.4 %
Health assistant, secondary school	3.1 %
Received in-service training	
On HIV/AIDS	58.5 %
On Focused ANC	31.3 %
Number of years as health worker (mean/median/range)	14.1/11.0/0–39
Number of years employed at this health center (mean/median/range)	6.4/3.5/0–29
Number of previous postings (mean/median/range)	1.6/1.0/0–7
Travel time between home and facility, in minutes (mean/median/range)	14.6/7.0/2–90

#### Availability and distribution of staffing

Providers commented on the lack of human resources at health centers, with some attributing increased queues for integrated antenatal services to the insufficient number of health workers. The average number of health care workers per health center was 24, with approximately 11 providing RCH services. Of those providing RCH services, 36 % were absent on the day the research team visited the health center due to annual or sick leave, training, work trips, or travelling to collect their salaries. While health centers in suburban and rural settings on average met MoHSW recommended staffing levels for most cadres, the urban health center exceeded the recommended staffing level for assistant medical officers, clinical officers, and enrolled nurses (Table 3). Yet when considering the larger population this urban health center needed to cover, it had on average lower levels of staffing per population size than several rural health centers.

Human resource challenges led some health centers to designate one day each week as the day for HIV testing and counselling, which separated HIV testing and counselling from routine RCH services with implications for access to services and stigma.

*The shortage of facility health care providers …* [the woman] *has come today expecting that she is going to finish everything, then you are telling her of the other day* [for the HIV test]*, she may not come… there are others who don’t come back.* (Provider 05–03, registered nurse)

As a result of integration, health workers were expected to perform additional tasks related to HIV testing and counselling during antenatal services (Table 2). If HIV testing couldn’t be performed at the first antenatal visit or if the test result were positive, providers had additional follow up tasks to perform after the antenatal visit. Health workers reported that the increased workload, lack of sufficient providers, and lack of integration of care and treatment were demotivating.

*More than 60* [women] *in a single day, and you are only two… it discourages a lot, when you find* [a] *positive* [test result]*, you have to take her direct to CTC and ask them to attend her so that you can continue with others. What we want is all services of the* [HIV] *positive* [women] *to be done at RCH*. (Provider 02–27, enrolled nurse)

### Provision of HIV counselling

**Fig. 3:**
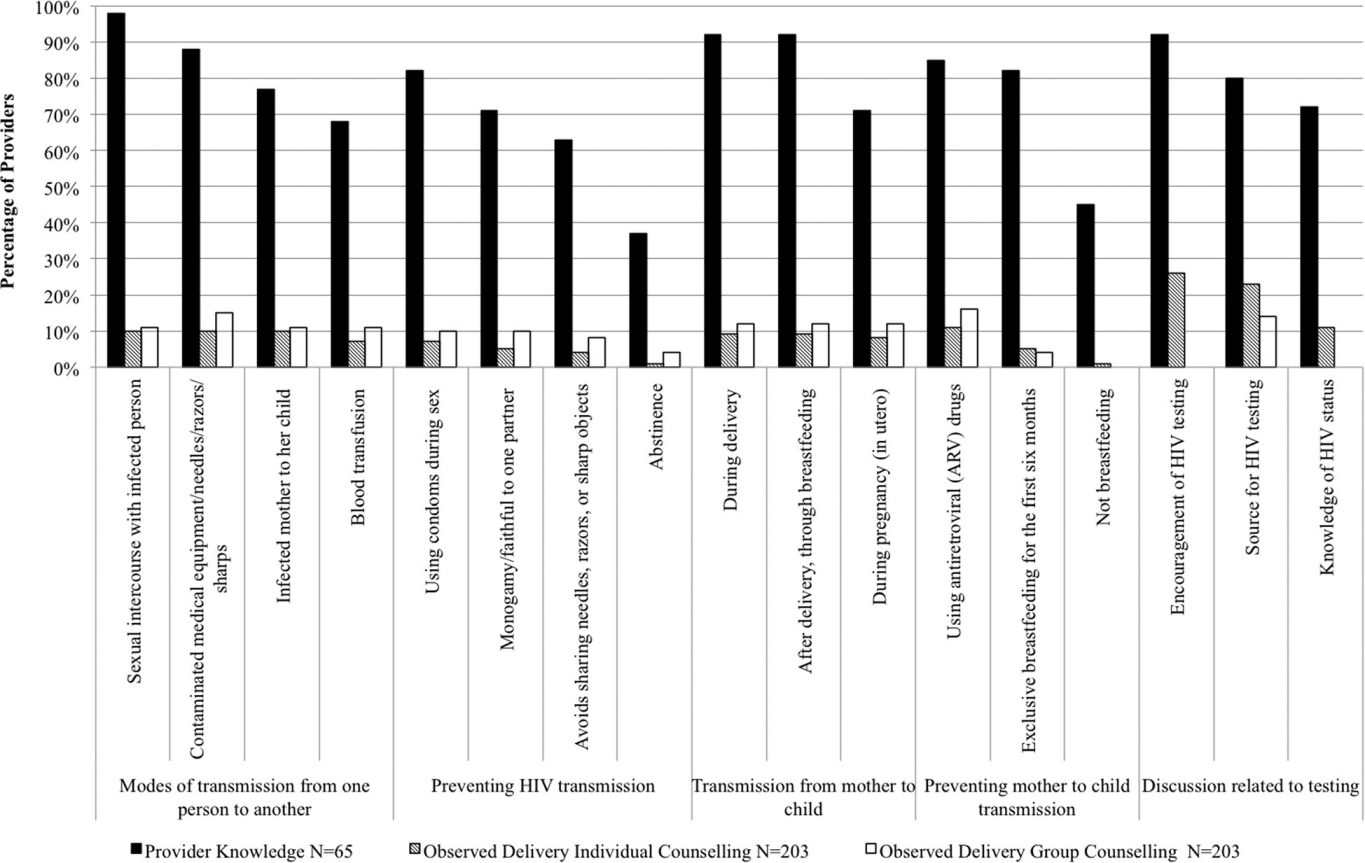
ANC provider knowledge and percent of observed counselling sessions with delivery of HIV- and ANC-related messages

At least two-thirds of health workers providing antenatal care recalled all specific modes of HIV transmission and three out of four ways of preventing HIV transmission. However, each mode of HIV transmission was mentioned in at most 10 % of observed individual antenatal sessions (20 out of 203 sessions, Fig. 3). Similarly, while 72 % or more of providers (47 or more out of 65 providers) could recall messages related to HIV testing without prompting, providers at only 11 % of individual counselling sessions (22 out of 203 sessions) were observed to have asked women whether they knew their HIV status, 26 % (53 out of 203 sessions) encouraged women to get tested for HIV, and 23 % (47 out of 203 sessions) gave information about where to access HIV testing services. Providers at just under a fifth (18 %, 36 out of 203) of antenatal counselling sessions were observed to have used job aides such as notes (Table 6). The MoHSW provides FANC job aides to all health centers providing antenatal care and PMTCT job aides to health centers where PMTCT services are available, to be used by providers who had received PMTCT training.

**Table 6:** Observed characteristics of ANC counselling sessions (*N* = 203)

Received group counselling^a^	50.0 %
Number of group counselling attendees (mean/median/range)	11.7/13.0/2–21
Main group counselling themes	
Maternal health during pregnancy	42.9 %
ANC messages	18.4 %
Newborn care	14.3 %
HIV/AIDS	11.2 %
Malaria	7.14 %
Maternal health, postpartum	6.12 %
Time between arrival at health center and being seeing by provider, in minutes (mean/median/range)	117.2/98.8/2–420
Duration of counselling in minutes	
Group counselling (mean/median/range)	18.6/15.0/10–33
Individual session (mean/median/range)	17.3/12.0/2–66
Provider used job aides	17.7 %

^a^Receipt of group counselling and individual counselling were not mutually exclusive. Some pregnant women received both

Analysis of the duration and content of antenatal counselling showed that providers had limited time with each patient (Table 6). Group counselling sessions covered a range of topics, including maternal health during pregnancy and after birth, antenatal care, newborn care, HIV/AIDS, and malaria. These group counselling sessions lasted a median of 15 minutes, ranging from 10 to 33 minutes. Individual counselling and clinical sessions lasted a median of 12 minutes, ranging from 2 to 66 minutes. The ranges in the length of time for counselling sessions, whether for group or individual counselling, suggested that despite FANC and PMTCT guidelines, substantial variability existed in counselling content. Health providers and patients indicated that the short time spent with each patient and long wait times for services were due to the lack of RCH providers (Table 6).

## Discussion

Infrastructure, supplies, and staffing are not adequate for HIV testing and counselling in antenatal care in health centers in rural districts of Morogoro Region, Tanzania. While half of the health centers had the physical space for service provision, observation of long queues raised concerns about adequacy of waiting areas and privacy and confidentiality of HIV test results and counselling. Unreliable supplies of test kits and essential drugs and equipment led to uneven implementation of integrated HIV testing and counselling, affecting quality of care and patient satisfaction, as well as provider-patient relations. Health workers perceived a shortage of staff, although uneven distribution may be more of a concern. In addition, high workloads may be resulting in short antenatal care sessions with truncated counselling messages despite high knowledge levels of providers. As facilities are upgraded from one level of the health system to the next, we found a large amount of heterogeneity in results across health centers, which did not correlate with health centers’ catchment area, catchment population, or administrative designation of urban, suburban, or rural settings. Structural inputs, such as infrastructure, supplies, and staffing, must be ensured as their lack of availability have dynamic effects on accessing and continuing care; confidentiality of services; time and quality of counselling; alongside the trust, reputation and morale of health workers. Taken together, these results suggest that while integration at the point of delivery through the merging of some services has potential, it still faces major challenges in meeting the proposed benefits that answer the “why” of integration: improved coverage, access, quality, and reduced cost that ultimately contribute to mitigating the impact of HIV/AIDS [19]. In the next section, we review each of these areas individually.

### Infrastructure

Enough physical space at health facilities was associated with higher odds of health workers conducting CD4 testing in an ART program in Zambia [36]. Higher infrastructure quality was also associated with higher PMTCT service coverage in health facilities in Cameroon, Côte d’Ivoire, South Africa, and Zambia, while not enough space, or lower quality infrastructure, negatively affected PMTCT services [37]. Inadequate space and overcrowding are barriers to confidential and private HIV counselling and testing and communication of results during care provision, which are key processes in the delivery of care and a major concern to patients [7, 38]. Integration without reorganization of routine service delivery and additional resources is not enough to compensate for and can lead to systemic deficiencies with regards to space and physical infrastructure. One study in Zambia found that while integration led to a reorganization of service delivery and improved management of services, integration did not resolve deeper infrastructural issues, including inadequate and inappropriately equipped space [39].

In Tanzania, the health centers where antenatal services are delivered were constructed 20 to 30 years ago before the era of HIV. As such, they lack privacy and adequate space for delivering quality antenatal care including PMTCT services. Over time, the population has outgrown the space at the health facilities, which has led to overcrowding and long wait times. The integration of PMTCT intervention in RCH services has increased the spacing challenge. Reorganization of the RCH clinics to create space for privacy and confidentiality is a minimum prerequisite for HIV testing and counselling. In addition, solutions for reducing the overcrowding of antenatal care services include increasing the number of clinic days, creating clinic shifts (i.e. morning and afternoon clinics), expanding service to the dispensary level, and encouraging women to start antenatal care at the dispensary level. In the long term, addressing the spacing challenge in health centers can only be done by working with district and municipal councils to support and prioritize substantial infrastructural expansion.

### Supplies

Health system constraints such as HIV test kit and drug shortages have been reported in Tanzania and other scaled-up PMTCT programs [40, 41]. Such supply stock-outs are a feature of supply chain issues in general and are not specific to HIV programs [42]. The strategy of using part of HIV funding to strengthen health systems in Kenya, Malawi, Zambia, and Mozambique has shown some success in ensuring adequate stock of supplies for integrated services and strengthening primary care more broadly [43–45].

Prior to our study, the Tanzania Service Provision Assessment Survey in 2006 found that only 24 % of all health centers offering anti-retroviral therapy had no stock-outs of the medicines in the six months preceding the survey [31]. Subsequently, in a study in southern Tanzania, providers attributed frequent stock-outs and delays in equipment repairs to lack of transportation, low fuel, and supplier issues [37]. The withdrawal of Bioline HIV test kits from circulation in Tanzania following a WHO recommendation due to concerns about Bioline’s quality could have also contributed to test kit shortages at health centers at the time of our survey [46]. To change the timeliness with which commodities are delivered and maintained in health facilities, the capacity of existing health workers in estimating the amount of stock and reordering needs to be improved. Autonomy to procure drugs from sources other than the MSD when the latter cannot respond should also be explored.

### Staffing

With HIV testing and counselling integrated into antenatal care, providers have added responsibilities that increase the time needed to conduct counselling sessions, which leads to long queues [44, 47, 48]. The additional workload also affects quality of care through health worker motivation. A study in Rufiji district in Pwani Region, Tanzania found that providers did not feel adequately trained for HIV testing and counselling [7]. In another study in Mbeya Region, Tanzania, providers reported that they felt disempowered and uninvolved in scaling up and sustaining antiretroviral therapy and that the care and management of HIV-positive pregnant women were outside their control [49].

The additional workload associated with integrated services led some facilities in this study to reorganize service provision so that some services, for example HIV testing, were provided only on specific days. Furthermore, health workers and pregnant women perceived that the lack of health care workers led to overcrowded health centers. These human resource constraints could limit and even reverse the gains of integration in increasing time savings, coverage, and access to services for pregnant women. Further involvement of health workers when implementing service delivery changes is critical. Initiatives that ensure supportive supervision, ongoing motivation and retention of staff, particularly in underserved areas, are important. Longer term changes include task-shifting measures [47] as well as improved ability to train and post health workers to address overall shortages.

### Provision of HIV counselling

Although providers report that HIV testing and counselling is an expected part of routine antenatal care services, this study found that counselling was uneven and remained below 50 %. Faced with long queues, providers limited the counselling messages they conveyed to women in the short amount of time they had during antenatal consultations. A study in eastern Uganda found that when providers limited counselling sessions after HIV testing, some pregnant women left the consultation with unanswered questions, leading to a missed opportunity for HIV prevention [50]. Our finding suggests that even with high levels of provider knowledge, benefits of integrated service delivery are limited if providers are unable to deliver elements of integrated care. Provider-initiated testing and counselling has led to overstretched providers and long wait times, which further disincentivize women from returning to health centers for routine antenatal services. Potential solutions include supportive supervision conducted on a regular basis and community-based initiatives that support HIV counselling, such as engaging HIV-infected mothers in mother-to-mother programs.

### Option B+

As global health policy moves toward strengthening HIV and RCH services, integration as a strategy is becoming more important. Some studies show that uptake of treatment is increased when antiretroviral therapy is integrated into antenatal care [51, 52]; however, a study in Malawi indicated that under Option B+, women who initiated antiretroviral therapy during pregnancy were five times more likely than those initiating treatment after staging or cell blood count to be lost to follow up [53]. In Tanzania, from a service delivery standpoint, linkages between RCH and CTC are still lacking even in the antenatal period, with many pregnant women dropping out after HIV testing and not connected to longer-term HIV care or follow up [45, 54, 55]. The Tanzanian MoHSW moved towards Option B+ in late 2013 and by 2014 began scaling up the program nationally. Thus far, challenges include lack of drug adherence among women who are not ill and high loss to follow up as well as emergence of drug resistance. One way to address high loss to follow up is to utilize community health workers and community home based care.

## Study limitations and strengths

This was a cross-sectional study, conducted during the harvest season. Clinics could be even busier during non-harvest periods than what the research team observed. With regards to the interviews, informant fatigue could have resulted from long in-depth interviews, which usually followed quantitative interviews. This fatigue was mitigated by rescheduling the interview with rest periods between the quantitative and qualitative portions of interviews. Rest periods ranged from half an hour to an entire day, so as to minimize the impact of research on interviewees’ other activities.

As a part of a larger evaluation of maternal and newborn services, we focused on results related to the supply-side capacity for HIV testing and counselling in antenatal care. Since this was integrated in 2007, our study design did not enable us to assess the effects of integration on service delivery. In addition, a facility’s capacity and readiness to deliver integrated care does not necessarily lead to actual delivery of integrated care [56]. Nevertheless, service delivery and its components are still critical aspects of integration, and by focusing on antenatal services, this study examined the key period during which women enter the MNCH spectrum of care and included tangible areas for improvement. The study also triangulated data across respondent types, investigators, and methodology to increase the validity and reliability of results. The results from this study are useful in examining challenges and gaps in integrated service delivery in the broader context of Tanzania’s national PMTCT scale up, especially as the country continues to integrate HIV care and treatment into maternal and child health services with Option B+.

## Conclusion

This study focused on supply-side dimensions of service delivery, namely structural inputs (infrastructure, supplies, and staffing) and certain key aspects of care provision (provider knowledge and delivery of counselling, time for counselling, and time spent waiting for care). We found critical deficiencies that undermine the integration of HIV testing and counselling services in routine antenatal visits in rural Morogoro, Tanzania. The space to ensure confidential testing and counselling, the supplies for HIV testing and prophylaxis for vertical transmission, the availability and distribution of human resources for health, and the delivery of counselling messages during routine antenatal care need improvement. With the shift toward Option B+, treatment and long-term management of HIV for women and children, previously part of separate CTC services, will be fully incorporated into RCH clinics. Existing deficiencies in structural inputs and processes of care delivery must be addressed so that the trend toward greater integration can succeed in improving service delivery and health outcomes across the continuum of care for mothers and newborns in Tanzania.

## Abbreviations

ANC: Antenatal care
AZT: Zidovudine
CTC: Care and treatment center
FANC: Focused antenatal care
HIV/AIDS: Human immunodeficiency virus and acquired immunodeficiency syndrome
JHSPH: Johns Hopkins School of Public Health
MNCH: Maternal, newborn and child health
MoHSW: Ministry of Health and Social Welfare
MUHAS: Muhimbili University of Health and Allied Sciences
PMTCT: Prevention of mother-to-child transmission
RCH: Reproductive and child health
SBMR: Standards-Based Management and Recognition
sdNVP: Single dose nevirapine
SPA: Service Provision Assessment
WHO: World Health Organization
3TC: Lamivudine

## Footnotes

1 Kilosa district has subsequently split into Gairo and Kilosa districts, but we report results according to their administrative designation at the time of data collection in 2012.
